# Multifaced Roles of HDL in Sepsis and SARS-CoV-2 Infection: Renal Implications

**DOI:** 10.3390/ijms22115980

**Published:** 2021-06-01

**Authors:** Alessandra Stasi, Rossana Franzin, Marco Fiorentino, Enrico Squiccimarro, Giuseppe Castellano, Loreto Gesualdo

**Affiliations:** 1 Renal, Dialysis and Transplantation Unit, Department of Emergency and Organ Transplantation, University of Bari, 70124 Bari, Italy; rossana.franzin@uniba.it (R.F.); mfiorentino84@gmail.com (M.F.); 2 Department of Emergency and Organ Transplant (DETO), University of Bari, 70124 Bari, Italy; e.squiccimarro@gmail.com; 3 Cardio-Thoracic Surgery Department, Heart & Vascular Centre, Maastricht University Medical Centre (MUMC), 6229HX Maastricht, The Netherlands; 4 Nephrology, Dialysis and Transplantation Unit, Advanced Research Center on Kidney Aging (A.R.K.A.), Department of Medical and Surgical Science, University of Foggia, 71122 Foggia, Italy; giuseppe.castellano@unifg.it

**Keywords:** lipid profile changes, dysfunctional HDL, sepsis, SARS-CoV-2 infection, acute kidney injury (AKI)

## Abstract

High-density lipoproteins (HDLs) are a class of blood particles, principally involved in mediating reverse cholesterol transport from peripheral tissue to liver. Omics approaches have identified crucial mediators in the HDL proteomic and lipidomic profile, which are involved in distinct pleiotropic functions. Besides their role as cholesterol transporter, HDLs display anti-inflammatory, anti-apoptotic, anti-thrombotic, and anti-infection properties. Experimental and clinical studies have unveiled significant changes in both HDL serum amount and composition that lead to dysregulated host immune response and endothelial dysfunction in the course of sepsis. Most SARS-Coronavirus-2-infected patients admitted to the intensive care unit showed common features of sepsis disease, such as the overwhelmed systemic inflammatory response and the alterations in serum lipid profile. Despite relevant advances, episodes of mild to moderate acute kidney injury (AKI), occurring during systemic inflammatory diseases, are associated with long-term complications, and high risk of mortality. The multi-faceted relationship of kidney dysfunction with dyslipidemia and inflammation encourages to deepen the clarification of the mechanisms connecting these elements. This review analyzes the multifaced roles of HDL in inflammatory diseases, the renal involvement in lipid metabolism, and the novel potential HDL-based therapies.

## 1. Introduction

Infection and inflammation induce marked alteration of the lipid and lipoprotein profile, leading to impairment of host immune response and worsen outcome [1]. Accumulating evidence has shown that viruses, bacteria, and fungal pathogens are able to manipulate host lipid pathways, modifying energy storage and production in cells, immune signaling, and tissue repair [1].

High-density lipoprotein (HDL) is a key component of circulating blood and mainly contains phospholipids, free cholesterol, cholesteryl ester, triglycerides, apolipoproteins, and other proteins. Besides its role in reverse cholesterol transport, HDL displays pleiotropic functions during inflammation and endothelial dysfunction, decreasing inflammatory signaling in immune effector cells and inhibiting endothelial response [2,3]. It is increasingly recognized that HDL can bind and neutralize viruses and toxic bacterial substances such as lipopolysaccharide (LPS) [4,5,6] and lipoteichoic acid [7]. The binding of LPS by HDL has been shown to protect animals from the toxicity of endotoxin [8,9]. Moreover, HDL could block certain viruses to penetrate cells, reducing tissue invasion [1,10].

Clinical studies have demonstrated that HDL levels drop by 40–70% during systemic inflammation, leading to a poor prognosis in septic subjects [11,12,13,14,15]. Moreover, low levels of HDL have been associated with increased risk of acute kidney injury (AKI) in the course of sepsis [16,17]. Renal function and plasma HDL are strongly related to each other as kidneys are implicated in the recycling of senescent HDL particles and their filtration function is associated with their levels and contents [18].

The new respiratory infectious disease, coronavirus disease 2019 (COVID-19), showed common features of sepsis pathogenesis, such as the dysregulated host immune response, alteration in serum lipids, endothelial dysfunction, and changes in the coagulation system [19,20]. In addition, some COVID-19 patients are admitted to the intensive care unit (ICU) developing acute respiratory distress syndrome (ARDS), acute cardiac injury, acute kidney injury (AKI), and shock [21,22,23].

Since the major contributors to mortality in septic patients are the hyperinflammation, the endothelial dysfunction, and low HDL is prognostic of worsen outcome, considering the similarities between sepsis and COVID-19 infection, it is reasonable that HDL replacement therapy has been a well sought-after area of systemic inflammatory syndrome therapies.

In this review, we discuss the multiple functions of HDL and its role in sepsis and SARS-COV-2 infection, summarizing the principal pathways involved and the new potential therapies.

## 2. HDLs Composition and Reverse Cholesterol Transport Function

HDLs are defined as a class of complex nanoparticles that consist of a broad variety of lipids and proteins [24]. Their structure includes a hydrophobic nucleus composed by triglycerides and esterified cholesterol and an external part with phospholipids and free cholesterol, associated with proteins named apoproteins [24].

The protein and lipid composition ratio depends on several physiological and pathological factors. The different protein and lipid cargo determines HDLs particles subdivision in five distinct classes: very large, large, medium, small, and very small HDL [24,25].

The principal pleiotropic function of HDL is Reverse Cholesterol Transport (RCT), leading the delivery of cholesterol from peripheral tissue to the liver where it is metabolized [26]. The cholesterol mobilization and transport are necessary to avoid its accumulation in cells, preventing cellular apoptosis [27,28]. This process starts with the biosynthesis of premature HDL, composed principally by ApoA-I, in the liver and small intestine [26]. Then, this premature HDL acquires phospholipids and free cholesterol through the interaction with ATP-binding cassette transporter A1 (ABCA1), principally expressed by macrophages and hepatocytes and becomes pre-β HDL [26]. The absence of a non-polar lipids core in pre-β HDL induces a disk-shaped structure, recognized as nascent HDL [26]. This nascent HDL quickly evolves toward mature HDL acquiring cholesterol, cholesterol ester, and triglycerides, and this remodeling process is catalyzed by a specific enzyme called lecithin–cholesterol acyltransferase (LCAT) [26]. Mature HDLs continue to acquire free cholesterol and apolipoproteins and exchange esterified cholesterol with triglycerides from other lipoproteins due to the action of lipid transfer proteins such as cholesteryl ester transfer protein (CETP) [26]. Then, these large HDLs return to the liver, bind the scavenger receptor class B type I (SR-BI), and facilitate cholesterol esters uptake, leading to their degradation by hepatocytes and their excretion into the bile [26]. This process is followed by the synthesis and release of a new premature HDL, enhancing a new cycle of RCT. There is a second mechanism for cholesterol degradation that is mediated by mature low-density lipoproteins (LDLs) that acquire cholesterol from HDLs by CEPT and are internalized by hepatocytes via the LDL receptor (LDL-R) [29].

The analysis of the HDL proteomic profile revealed a large protein content mainly characterized by ApoA-I, other major apolipoproteins including ApoA-II, Apo-CI/CII/CIII, and remodeling HDL enzymes and minor crucial proteins such as complement and coagulation regulatory factors, protease inhibitors, and acute-phase proteins that reflect HDL involvement in distinct pleiotropic functions [30]. The role of ApoA-II is still not clear in inflammatory diseases and some studies reported pro-inflammatory and anti-inflammatory role, respectively [31,32]. Indeed, Furlaneto et al. demonstrated that ApoA-II hampered neutrophil activation, reducing oxidative burst and IL-8 synthesis [32]. Another study showed that ApoA-II could increase monocyte response to LPS, thereby amplifying inflammatory reaction [31]. A minor apolipoprotein found in HDL is the apolipoprotein M (ApoM) that transports the Sphingosine 1-phosphate (S1P), a lysophospholipid mediator involved in several physiological functions such as cellular proliferation and survival, endothelial nitric oxide synthase (eNOS) activation, and inhibition of inflammation and endothelial dysfunction [33]. Recently, Kurano et al. demonstrated that ApoM was not only a carrier but it regulated and increased S1P content, reducing acute lung injury (ALI) and incidence of mortality in the endotoxemic mouse model [34].

Another functional protein, discovered by mapping HDL protein content by two-dimensional gel electrophoresis and mass spectrometry, is the alpha-1 antitrypsin (AAT), a natural inhibitor of serine proteases, chiefly neutrophil elastase but also chymotrypsin, cathepsin G (CathG), proteinase 3 (PR3), and thrombin [35]. AAT deficiency is strongly associated with an increasing risk to develop pulmonary emphysema due to lung damage caused by neutrophil elastase activation. To prevent pulmonary aggression by elastase, infusion of purified AAT proteins has been used as treatment not only in AAT deficient conditions but also in other inflammatory diseases [36].

The lipid composition of HDL includes different species among phospholipids, free and esterified cholesterol, sphingolipids, and triglycerides that could protect cells from infection. Indeed, Bricarello et al. found that gangliosides fixed in reconstituted HDLs (rHDLs) could bind cholera toxin, limiting its detrimental effects [37]. As previously indicated, S1P is an important player of inflammatory response and endothelial dysfunction [33]. In particular, S1P induces eNOS pathway activation and inhibition of monocyte chemotactic protein-1 (MCP-1) synthesis in endothelial cells by binding S1P-receptor-1 [38,39,40,41]. Several studies reported its involvement in regulating endothelial permeability, cytokines release, and inflammatory response in sepsis context [42,43]. In a single-center prospective-observational study, Winkler et al. showed a strong inverse correlation between Sequential Organ Failure Assessment (SOFA) scores and serum-S1P levels in septic patients, underlying the potential role of lipids in systemic inflammatory diseases [42].

## 3. HDLs Pleiotropic Functions

**Figure 1:**
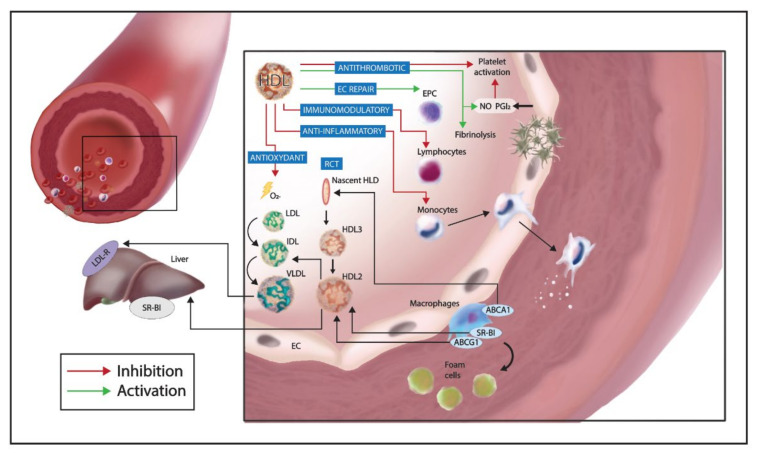
Schematic representation of HDL multi-protective mechanisms. The principal function of HDL is the reverse cholesterol transport (RCT). In atherosclerotic lesions, HDL induces cholesterol efflux from macrophages, avoiding foam cells formation. The process starts with premature HDL (Nascent HDL) that interacts with ATP-binding cassette transporter A1 (ABCA1), expressed on macrophages, and acquires phospholipids and free cholesterol. This nascent HDL evolves in mature HDL (HDL3) that further acquires cholesterol, cholesterol ester, and triglycerides via specific enzymes and interacting with other lipid transporters such as scavenger receptor class B type I (SR-BI) and ATP Binding Cassette Subfamily G Member 1 (ABCG1). This large HDL (HDL2) turns to the liver and after binding SR-BI, cholesterol esters are internalized and degraded and excreted into the bile. The second mechanism for cholesterol degradation is mediated by mature low-density lipoproteins (LDLs) that uptake cholesterol from HDL2 mediating cholesterol internalization by hepatocytes via the LDL receptor (LDL-R). Other important functions of HDL include anti-thrombotic activities (increased expression of nitric oxide [NO] and prostacyclin [PG12] in endothelial cells with reduced platelet activation and augmented fibrinolysis), anti-inflammatory effects (reduced monocytes activation), immunomodulatory properties (lymphocytes anergy), and anti-oxidant effects (prevention of LDL oxidation).

Besides their role in RCT, HDLs are involved in modulation of oxidative stress, inflammation, immune response, coagulation activity, and endothelial dysfunction [44] (Figure 1). These protective effects have been studied most intensively in physiological conditions and their impairment is found in a pathological context such as dyslipidemia, atherosclerosis, and infection diseases [44]. Indeed, numerous studies commonly referred to HDLs as principal players in infection field, underlying their role in neutralizing detrimental activity of bacteria, viruses, and parasites.

### 3.1. Scavenger Effects Against Bacterial and Viral Infection

In the context of bacterial infections, HDLs bind the major components of Gram-negative and Gram-positive bacteria promoting their inactivation and clearance [45].

Inherent to Gram-negative infections, LPS, the principal component of the outer bacterial membrane, is recognized by its specific receptor Toll Like Receptor-4 (TLR-4) expressed in immune cells and nonprofessional cells [45]. Upon LPS binding to TLR-4, cells activate downstream inflammatory signaling, leading to pro-inflammatory mediators synthesis such as TNF-α and IL-6, hampering cellular damage [46].

Circulating LPS preferentially binds HDL particles and this association seems to be due to lipid A, the lipid component of endotoxin [47]. This binding is mediated by Phospholipid Transfer Protein (PLTP) and CEPT that share sequence homology with the principal LPS serum carrier proteins, named LPS-binding protein (LBP) and bactericidal/permeability-increasing protein (BPI) [48,49]. PLTP is able to bind and extracts LPS monomer from the outer bacterial membrane and mediates its transport to HDL, alone or in combination with LBP, attenuating LPS/TLR-4 signaling [49]. Interestingly, Gautier T. et al. demonstrated in deficient mice of the PLTP gene that this enzyme is not only involved in the metabolism of HDL, but it is pivotal in LPS clearance, accelerating the so-called ‘reverse LPS transport’ pathway [50]. In addition, another study highlighted the role of ApoA-I in preventing endotoxin recognition by TLR-4 and inhibiting pro-inflammatory pathways in immune and parenchymal cells of endotoxemic rats [51]. Lipids emulsion could neutralize LPS effects, in particular, it has been shown that the phospholipids content increased LPS binding, whereas cholesterol and triglyceride exerted opposite effects [52]. Whatever the mechanism, an increased number of studies demonstrated the ability of HDL to counteract Gram-negative infection and alterations in HDL composition were associated with an impaired response to infection [53].

The effects of HDL were also observed in Gram-positive bacterial infection, for their ability to neutralize the outer membrane component, called lipoteichoic acids (LTA), which induces activation of pro-inflammatory response [7,54]. In particular, LTA induces endothelial dysfunction and the release of inflammatory cytokines by immune cells through TLR-2 binding [7,55,56]. Jiao Y. et al. demonstrated the effects of ApoA-I in mediating LTA neutralization with inhibition of inflammatory response and decrease of lung injury in mouse model of sepsis-induced-ALI [57].

During viral infection, lipoprotein levels and composition are significantly modified. Hepatitis C virus is present in the bloodstream associated with triglyceride-rich lipoproteins and HDL could mediate viral entry via SR-BI [58]. Comparably, Dengue virus shows increased affinity to ApoA-I which facilitates virus infection into surrounding cells [59]. Therefore, SR-BI might be necessary for efficient binding of viral particles and thus viral entry into host cells. In the human immunodeficiency virus (HIV) infection, HDL metabolism is modified, and these alterations seem to be associated with disease progression [45].

It is not clear if HDL is good or bad for viral infection. HDL could exert also antiviral activities, by binding and neutralizing viral DNA and RNA [60]. Current evidence suggests that viral inactivation could be induced by ApoA-I interference with viral entry, or inhibition of viral fusion with host cells [61]. Several experimental studies demonstrated that infusion of ApoA-I mimetic peptide could reduce lung damage in a mouse model of influenza A infection [62,63]. Therefore, therapeutic approach finalized in the use of ApoA-I could fight influenza A-induced pneumonia and systemic inflammation, reducing alveolar cells damage and alveolar macrophage response [62,63]. Considering these results, the use of HDL in viral infection diseases with lung tropism could be addressed in further studies.

### 3.2. Modulation of Inflammation and Immune Response

The anti-inflammatory activity of HDL has been well-documented during the atherosclerotic process and sepsis disease. HDLs have been shown to stimulate Nitric Oxide (NO) release from endothelial cells, decrease leukocyte adhesion, promote RCT, modulate cytokines production, and act as an antioxidant and an anti-thrombotic agent (Figure 1) [64].

During an inflammatory response, HDL and ApoA-I alter adhesion molecules expression on leukocytes and endothelial cells, inhibiting leukocytes adhesion, rolling, and infiltration, and consequently reducing inflammatory process [65]. Moreover, ApoA-I modulates the synthesis and release of pro-inflammatory cytokines and reactive oxygen species (ROS) by immune cells [66]. For example, in atherosclerotic lesions, activated macrophages and neutrophils release several cytokines, ROS, and proteases, all of which induce inflammatory injury [66]. In particular, cytokines promote the recruitment of other circulating immune cells to the lesion and ROS oxidate circulating LDL, promoting the influx of oxLDL into macrophages, leading to foam cells formation in subendothelial plaques [66]. Proteases induce degradation of the extracellular matrix and the release of inflammatory fragments that amplify local damage. In this context, the enrichment of serine proteases inhibitor (SERPINs) in HDL cargo allows HDLs to inhibit and regulate several proteases involved in inflammation, coagulation, and complement system, preventing inflammatory disease progression [66].

Among potent proteases involved in atherosclerosis, neutrophil elastase (NE) could be a potential modifiable target of HDL activity. The increased activity of NE is associated with unstable plaque morphology and higher cardiovascular risk and mortality [67]. Therefore, the inhibition of this protease could be an efficient strategy to reduce subendothelial plaques. The natural inhibitor of NE is alpha-1 antitrypsin (A1AT), a serpin inhibitor, principally produced by the liver and significantly increased in atherosclerosis process [68]. Since A1AT could not across vascular endothelial cells of large arteries, the association of HDL and A1AT may provide an alternative mechanism to transfer this inhibitor into the inflammatory lesions [68,69]. Duckers J.M. et al. reported that A1AT deficient patients presented reduced vessel elasticity and elevated risk to develop cardiovascular disease [70]. Interestingly, Moreno et al. showed that in a mouse model of emphysema, injection of A1AT complexed with HDL reduced neutrophils and macrophages amount and IL-6, MCP-1, and TNF-α levels in bronchoalveolar lavage fluid and prevented lung damage preserving pulmonary function [36].

HDLs influence immune cells response by modifying cholesterol amount in their plasma membrane and thus suppressing the activation of key receptors [71]. Indeed, HDL and ApoA-I influence CD11b expression on monocytes, reducing adhesion on endothelial cells [72]. Other in vitro studies demonstrated that macrophages in presence of HDL acquired the anti-inflammatory M2 phenotype, decreasing TLR-2 expression and increasing the activating transcription factor 3 (ATF3) synthesis [73]. Moreover, ApoA-I downregulates TLR-4 expression on endothelial cells, preventing the activation of the downstream proinflammatory signaling [74]. Dendritic cells are also influenced by increased HDL levels that cause a reduced expression of major histocompatibility complex II (MHC II) and IL-2 synthesis, thus inducing lymphocytes anergy (Figure 1) [75].

Another important role of HDL consists in the regulation of the complement system [76]. As well known, the complement pathway has a central role in innate immunity and involves a cascade of proteolytic events that lead to the synthesis of proinflammatory mediators and the terminal attack complex (C5-b9 complex) that induces cell lysis [76]. Complement activation is not only involved as a mechanism of protection against infection but there are several studies that underline its role in cardiovascular disease [77]. Proteomic analysis has revealed the presence of complement system proteins associated with HDL in physiological and pathological conditions. In particular, HDL of healthy subjects was found associated with C4a, C4b, and C9, whereas HDL of patients with coronary artery disorder presented higher composition of C3 and C4 factors [78].

HDLs includes several inhibitors of complement system in their cargo. In particular, Clusterin is associated with ApoA-I and can inhibit the cytolytic activity of C5-b9 complex [79]. ApoE can bind Factor H and regulates alternative pathway activation [80]. Both ApoA-I and ApoA-II-block C5-b9 assembly on endothelial cells, preventing complement-induced cell death [81].

Even if HDL showed anti-inflammatory activities, its composition could undergo alterations during inflammation, causing the loss of beneficial effects. In an inflammatory context, HDL acquires acute-phase proteins, such as serum amyloid A (SAA) and loses ApoA-I [82,83]. Therefore, HDL presents SAA up to 80% protein composition and has defective anti-inflammatory functions, antioxidant capacity, and is less effective in RCT and endothelial repair [82,83].

### 3.3. Prevention of Endothelial Dysfunction

Most of the pleiotropic effects of HDL are targeted to the endothelial layer, in order to preserve endothelial cells function [64]. HDL particles are able to directly interact with endothelial cells by binding endothelial apical receptors such as SR-BI, ABCA1, ABCG1, and ecto-F1-ATPase, inducing HDL internalization and activation of intracellular signaling pathway [64].

In particular endothelial SR-BI signaling in response to HDL triggers the activation of the downstream eNOS pathway, leading to NO production and vascular protection [64]. Moreover, Zhang et al. showed that SR-BI signaling induced an increased expression of cyclooxygenase 2 and PGI_2_ in endothelial cells, reducing platelet activation and thrombosis [84] (Figure 1).

As previously underlined, ABCA1 mediates cholesterol efflux from macrophages, preserving subendothelial space from plaques generation [85]. This receptor is also expressed by endothelial cells and is upregulated by increased LDL levels [86] (Figure 1). Accordingly, in an animal model of hypercholesterolemia, endothelial cells increased ABAC1 expression on their apical membrane, probably to accelerate cholesterol efflux [87].

Even if ABCG1 has high sequence similarity with ABCA1 and is involved in cholesterol efflux in macrophages, its expression on endothelial cells is not correlated to cholesterol transport [88]. Terasaka et al. reported that increased expression and activation of ABCA1 preserved NO synthesis and secretion by endothelial cells, leading to vascular protection in mice fed with a high-cholesterol diet [89].

Ecto-F_1_-ATPase is an enzymatic complex expressed by hepatocytes that promotes endocytosis of HDLs via non-conventional receptors [90]. The expression of Ecto-F_1_-ATPase on the surface membrane of endothelial cells seems to be involved in HDL transport and in inhibition of apoptosis [91].

Another important function of HDL consists in reducing expression of endothelial adhesion molecules in inflammatory conditions. Several studies reported that HDL inhibits pro-inflammatory cytokines such as TNF-α, reducing the expression of endothelial adhesion markers such as intercellular adhesion molecule-1 (ICAM-1), vascular cell adhesion molecule-1 (VCAM-1), and E-selectin [92]. Therefore, the regulation of endothelial phenotype by HDL reduces the recruitment of leukocytes and the incidence of thrombosis [92]. Indeed, HDLs possess also anticoagulant properties, reducing tissue factor (TF) expression on endothelial cells [93]. TF is involved in endothelial pro-coagulant activity and its decrease is associated with reduced fibrin deposition and diminished platelets activation [93]. Moreover, HDLs induce PGI expression in endothelial cells, preventing thrombotic complications [94].

HDLs have been shown to preserve endothelial viability, counteract oxLDL and TNF-α detrimental effects [95]. Indeed, de Souza et al. reported that HDL may display anti-apoptotic effects, reducing intracellular ROS generation and the activation of apoptotic mitochondrial signaling [95]. Moreover, the authors observed that anti-apoptotic functions are mainly associated with ApoA-I expression [95].

Endothelial dysfunction is a common hallmark of systemic inflammatory disease and CVD [96]. Several studies have evaluated the effects of HDL therapeutic approach to limit endothelial dysfunction in different pathological settings [96]. Further investigation is needed to better identify the perfect cargo of HDL to amplify their pleiotropic functions, particularly at the endothelial level.

### 3.4. Anti-Coagulant Properties

The anticoagulant effects of HDL are correlated to their lipid and protein composition that could influence the coagulation pathway at several steps modulating protease activators and inhibitors [76]. As well known, coagulation is the principal mechanism to avoid blood loss at a site of injury and is mediated by several proteolytic events that lead to the activation of thrombin and the formation of insoluble clots [97].

First studies showed that HDLs preserved endothelial integrity, preventing endothelial apoptosis, inhibiting molecule adhesion expression, inducing NO production, and thus blocking coagulation cascade activation at vascular endothelium [64]. As previously reported, the protective effects on endothelial viability are principally associated to S1P that possess anti-apoptotic functions [64].

Additional anti-coagulant properties regard the direct effects of HDL proteins and lipids on coagulation cascade. ApoH has been implicated in the regulation of coagulation, promoting inhibitory effects on one side [98] and pro-coagulant mechanism under certain conditions [99]. ApoH, also known as beta-2 glycoprotein 1, has been found to reduce the activation of the contact phase system of the intrinsic pathway and platelet aggregation by inhibiting platelet prothrombinase activity [98]. However, Mori T. et al. showed that under certain physiological conditions, it competitively inhibited the binding of activated protein C (APC) to the phospholipid surface, facilitating coagulation cascade [99].

Otherwise, ApoA-I shows anti-coagulant properties and attenuates activation of coagulation cascade by binding and sequestering anionic phospholipids that are expressed on the surface membrane of injured cells [100]. Anionic phospholipids are also found in circulating microparticles released by activated platelets, leucocytes, and endothelial cells [101]. During inflammation and vascular dysfunction, microparticle numbers increase and several studies demonstrated their involvement in coagulation process, cells intercommunication, and intracellular signaling [101]. Several enzymes, in particular PLTP, are involved in phospholipids transport from microparticles to either ApoA-I or apolipoprotein B-containing HDL, inducing a strong reduction of coagulation cascade, by decreasing the surface area for the activation of the prothrombinase complex [100].

The antithrombotic effect of HDL is also mediated through the interaction between HDL and the serum protein C [102]. Indeed, HDL and ApoA-I have been reported to significantly enhance APC-induced FVa degradation, resulting in the inhibition of coagulation cascade and reducing the risk of thrombosis [103].

Recently, Chung W.D. et al. showed the effects of HDL in interfering with von Willebrand factor (VWF) self-association on the endothelial surface in a mouse model of moderate thrombotic microangiopathy [104]. In physiological conditions, vWF is synthetized and secreted by activated endothelial cells and these monomers aggregate to facilitate platelet adhesion and repair endothelial barrier. During intense inflammatory conditions such as sepsis, ADAM Metallopeptidase With Thrombospondin Type 1 Motif 13 (ADAMTS13), the enzyme that provides to remove vWF aggregates is inhibited, thus this process becomes uncontrolled, leading to the formation of occlusive thrombi in the small blood vessels [105]. In this study, mice treated with HDL presented reduced platelet adhesion and thrombocytopenia [104]. Moreover, the authors analyzed serum levels of ApoA-I in patients with sepsis or thrombotic thrombocytopenic purpura (TTP), comparing to the healthy group. They observed that both groups presented reduced levels of ApoA-I and increased vWF adhesion [104].

Another important consideration about HDL protection from thrombosis is related to its ability to increase clots permeability [106]. In a study of 136 apparently healthy subjects, Ząbczyk M. et al. determined plasma concentration of ApoA-I and HDL and correlated these values to clots permeability, observing that higher HDL concentration reduced the risk of thrombosis and were associated to lower clots density [106]. This connection could be explained via the interaction between HDL and plasminogen. This binding could facilitate the cleavage of plasminogen into plasmin by several proteases presented in HDL cargo such as kallikrein [106]. Plasmin is the principal proteases in the fibrinolysis pathway, and it mediates fibrin clots degradation. Therefore, HDL could be a suitable platform for plasminogen and kallikrein interaction, facilitating fibrinolytic pathway and reducing the risk of thrombosis in several pathological context.

HDL lipid cargo has been implicated in regulation of coagulation cascade. Negatively charged lipids attract and bind circulating thrombin, facilitating the interaction with functional modulators that inhibit thrombin activation and clots formation [76]. This process could be translated also to other components of the coagulation pathway [76]. Indeed, HDL assemble and inhibit TF and FVIIa, preventing activation of FX of the extrinsic pathway [107].

Another anti-coagulant of FX activity is the antithrombin-III, whose activity during blood clotting has been correlated to serum lipids levels. Accordingly, Winter J.H. et al. measured plasma and serum antithrombin levels by functional and immunological assays in normal and atherosclerotic subjects and observed that anti-thrombin activity was positively correlated to HDL levels [108]. Until now, the functional correlation between HDL and anti-thrombin has not been yet reported.

## 4. Sepsis Pathogenesis and Lipid Profile Changes

Sepsis is a complex disease characterized by a systemic inflammatory response that arises during infection. Gram-negative bacteria are considered the common pathogens involved in sepsis disease [109]. The host response includes pro-inflammatory and anti-inflammatory mechanisms that are necessary for pathogen clearance; in this context, they could become impaired and lead to systemic organ damage [110].

There is a strong link between infections and sepsis occurrence in ICU patients. Indeed, sepsis is still a great health issue in ICU and the high risk of mortality, and a lack of efficient therapies are still unacceptable [111]. Therefore, more efforts have to be addressed to better understand the pathogenesis of this disease and discover novel targeting candidates for innovative therapeutic approaches [111].

One of the principal mechanisms occurring in sepsis pathogenesis is the endothelial dysfunction [109,112]. Several events have implicated the endothelial dysfunction, such as the direct interaction with bacterial components, the release of pro-inflammatory cytokines by pathogens-activated immune cells and pro-coagulant mediators, and the increased leukocyte adhesion [112]. All these processes have a great impact on endothelial behavior, causing the loss of their physiological function and the acquirement of dysfunctional phenotype [112,113,114,115,116]. Since multiple mechanisms are involved in endothelial damage, therapeutic approaches that target a single mechanism could have limited effects [116]. Therefore, other investigations have to be carried out to identify potential multi-protective mediators that could regulate a broad spectrum of events that occurred in sepsis pathogenesis.

Several studies have reported that HDLs are endogenous particles with multi-protective effects and are able to counteract endothelial dysfunction, induce bacterial components detoxification, regulate immune response, and decrease pro-coagulant activities [2]. Most clinical observational studies reported significant reduced levels of lipids and lipoproteins, in particular, HDL amount, in critically ill patients and especially septic patients [117]. This decrease was correlated to marked inflammatory response and worsen outcome. Indeed, Chien et al. observed that low HDL levels on day 1 of severe sepsis predicted adverse clinical outcomes [12]. Accordingly, Barlage S. et al. analyzed 151 patients with severe sepsis and reported that decreased levels of ApoA-I were independently associated with 30-day-mortality [118]. Recently, an interesting study demonstrated that rare missense variant in Cholesteryl Ester Transfer Protein (CETP) gene induced a significant decrease in HDL amount and higher risk of mortality and more organ failure [119]. CETP is an important protein for HDL levels and its decrease could be associated with clinical prognosis and outcomes [119]. Accordingly, Trinder et al. performed a meta-analysis of seven cohorts examining the effects of a gain-of-function variant in CETP (rs1800777, p.Arg468Gln) on 28-day sepsis survival. They found a strong correlation between CETP activity and survival and also demonstrated in a humanized mouse model that CEPT inhibition preserved HDL levels and could improve outcome in septic subjects [120].

However, other studies did not report any association between decreased HDL level and reduced survival [11,121]. Overall, low HDL levels are described as good biomarkers of sepsis progression and organ damage.

During inflammatory diseases, HDL levels drop down and this decrease could be the results of several mechanisms [45]. Indeed, augmented HDL consumption, decreased HDL biosynthesis for liver damage, and the increased uptake by SR-BI could explain this significant decline [45]. Moreover, endothelial dysfunction could facilitate the loss of HDL from the blood compartment [11].

Other mechanisms correlated with HDL decrease are associated with proteome and lipidome remodeling of HDL particles. During sepsis, HDL lipidome is significantly altered and this modification results in increase of triglycerides and decrease of phospholipids. Recently, Curcic et al. showed that changes in HDL composition with lysophosphatidylcholine enrichment through increased secretory phospholipases A2 (sPLA2) activity suppressed platelet activation and thrombosis under acute and chronic inflammatory process [122]. Moreover, in a prospective observational study of 74 ICU patients, the authors measured lysophosphatidylcholine serum levels and observed a strong correlation between decreased levels and risk of 28-day mortality in severe septic patients [122]. Therefore, serial measurements of lysophosphatidylcholine could be a good predictor of 28-day mortality in critically ill patients with severe sepsis [122].

Remodeling in protein composition is also reported in a sepsis setting [11]. During infection and inflammatory response, SAA proteins increase and replace ApoA-1 in HDL particles. These dysfunctional HDL decrease their affinity to LCAT, CETP, or paraoxonase 1 (PON1), causing impairment of RCT and loss of their ability to regulate inflammatory response and coagulation [11]. Recently, Sharma et al. analyzed septic patients’ proteome profiles, confirming changes in apolipoprotein composition with increased SAA content [123,124]. Several studies underline the association of dysfunctional HDL with poor outcomes in sepsis disease [125,126,127,128].

As discussed above, HDLs exert multi-protective functions from neutralization of pathogens to prevention of exacerbated immune response and endothelial dysfunction [3]. Differently from monoclonal antibodies against LPS [129], HDLs are able not only to bind endotoxin but also to mediate its clearance by SR-BI [9]. Preclinical studies reported that endotoxemic or CLP mice deficient in SR-BI or HDL presented decreased LPS clearance and developed severe inflammatory disease [8,9,130]. Therefore, HDL provides LPS uptake and removal by SR-BI and promotes more protection compared to antibodies therapies against LPS.

Other studies demonstrated that also LTA was associated with HDL in blood circulation during Gram-positive infection [131].

During host response to infection, macrophages are considered the principal immune effectors, responsible for the elevated increase of circulating cytokines and consequent vascular damage [132]. Accumulating evidence indicates that HDL is a key regulator of macrophages response in inflammatory context [133,134]. Indeed, HDLs enhance the cholesterol efflux from macrophages, decrease their activation, and upregulate the transcriptional factor ATF3 which downregulates the expression of inflammatory mediators, ameliorating sepsis pathogenesis [73]. In addition, the decreased response of macrophages reduced endothelial dysfunction. Moreover, HDLs enhance eNOS activation via SR-BI binding on endothelial cells and prevent the acquirement of prothrombotic phenotype, suppressing TF and adhesion molecules expression [135].

In conclusion, the strong inverse relationship between low HDL and systemic inflammatory disease confers to HDL a key role in sepsis pathogenesis as a good biomarker and therapeutic target.

## 5. Kidney Involvement in Sepsis Disease: From HDL Target to Modulator

Acute kidney injury (AKI) is a common event in ICU patients, with an estimated incidence of >50% [136]. Furthermore, increasing AKI severity is associated with higher risk of mortality [137]. Sepsis is defined as the major cause of AKI [138], accounting for 45% to 70% of cases, and approximately 25% of sepsis is of intra-abdominal origin [139].

Early diagnosis of AKI in the setting of sepsis is important in order to provide optimal treatment and avoid further kidney injury [140]. Therefore, the use of injury or stress biomarkers, in addition to the clinical assessment of renal function, is required [141,142]. Inflammation appears to play an important role in sepsis-related kidney injury. Interleukin-6 (IL-6) has been described as predictive of AKI, independently of hypotension (e.g., mean arterial pressure, dosage of vasopressors) [143].

Since HDL levels significantly declined in severe sepsis and septic shock, several studies evaluated HDL impact on renal damage as a possible predictive biomarker and therapeutic target [144]. In a blinded, observational cohort study, the authors analyzed HDL amount in 200 adult patients with suspected sepsis at the time of admission [12]. They observed that HDL concentration is a prognostic factor to establish an early efficient therapy to avoid disease progression, multi-organ dysfunction, and renal damage [12]. In another observational small study of 180 patients with clear signs of infections, the authors demonstrated that low HDL levels are associated with increased risk of AKI onset and decreased glomerular filtration rate (GFR) [16]. These results suggest that HDL levels may accurately predict the development and the stages of AKI like serum creatinine levels for the Kidney Disease Improving Global Outcomes (KDIGO) index [16]. Interestingly, low levels of HDL in the early phase of sepsis could represent predictors of worsening renal function at three to twenty-four months after hospital discharge of septic patients with a dyslipidemia profile [16]. These results underline the key role of HDL in affecting renal function and disease progression.

In addition, polymorphisms of genes involved in HDL metabolism have been found strongly associated with increased risk of AKI during sepsis. Accordingly, Genga K.R. et al. retrospectively compared two cohorts of septic patients and demonstrated that one genetic variant in the CETP gene, rs1800777 (allele A), determined low levels of HDL and an increased risk to develop sepsis-related AKI [145].

Most of the current understanding of HDL involvement in sepsis pathogenesis and AKI have been revealed from animal models and in vitro studies. For example, Guo L. et al. demonstrated that higher expression of ApoA-I in transgenic mice increased survival and limited sepsis and renal damage compared to wild type mice [8,9]. HDL might provide renal protection through several mechanisms including pathogens detoxification via SR-BI internalization. As discussed above, SR-BI is expressed by immune cells, endothelial cells, and parenchymal cells such as hepatocytes and renal tubular cells and mediates the cholesterol efflux to circulating HDL. In addition, pathogens molecules as LPS and LTA could bind HDL in the bloodstream and could be transferred to SR-BI for hepatic clearance. Decreased serum levels of LPS or LTA induced lower activation of TLR-4 and TLR-2 respectively, in renal parenchymal cells [7,131,146,147]. Since both TLR-4 ad TLR-2 activation is associated with increased renal damage [148,149], it is obviously that HDL levels ameliorate renal function, avoiding tubular damage, interstitial inflammation, and cytokines release.

Endothelial dysfunction is a hallmark of sepsis-associated AKI [150]. In inflammatory conditions, endothelial cells increased inducible nitric oxide synthase activity (iNOS) and decreased eNOS activation [151], leading to vascular impairment and renal parenchymal damage [150]. Increased levels of HDL could activate the eNOS pathway, reducing adhesion molecules expression and leucocytes activation and neutrophil infiltration, assuring reduced renal damage [64].

**Figure 2:**
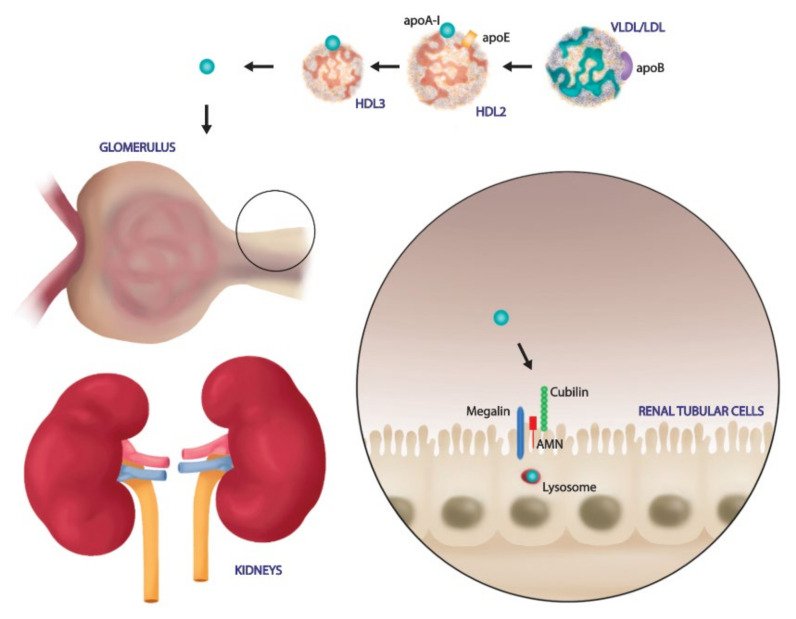
Renal HDL catabolism and transport. Senescent circulating HDLs are filtered in the glomerular capillaries. Renal tubular cells bind pre-B HDL particles or lipid poor ApoA-I via the Cubulin-amnioless complex and Megalin. HDL dissociates from tubulin in their endocytic vesicles and HDL is degraded into lysosome.

Renal tubular cells are not only the principal target of renal dysfunction, but they are directly involved in HDL modulation [152]. In the physiological condition, the glomerular filtration barrier is intact and prevents passage of large molecules such as mature HDL. Only lipid poor ApoA-I, ApoA-I, ApoA-IV and enzymes such as LCAT cross the filtration barrier. Then, renal tubular cells express on their surface membrane specific receptors to bind and internalize these particles. In particular, pre-β HDL and lipid poor ApoA-I bind to the cubulin amnion less complex on the surface of renal proximal tubule cells and are endocytosed; this process is accelerated by the binding of another membrane protein, megalin, that is essential for renal proximal tubule reabsorption [152]. After endocytosis, HDL could be degraded in tubular lysosome or transcytosed into intratubular lumen in order to be released into systemic circulation (Figure 2).

In early stages of renal dysfunction, the GFR is impaired and increased levels of Apo A-IV and LCAT are found in urines [153,154]. Certainly, the appearance of tubular damage determines reduced tubular reabsorption function and catabolism, contributing to loss HDL and its components. Accordingly, Aseem O. et al. reported that cubulin deficiency caused impaired renal transcytosis and consequent decreased serum levels of albumin, ApoA-I and HDL3 [155].

Moreover, kidneys can also affect HDL metabolism, influencing extrarenal synthesis and metabolism of HDL components [156]. For example, nephrotoxic syndrome induces a significant reduced expression of hepatic lipase in liver parenchyma, causing decreasing uptake of HDL triglycerides and phospholipids, increasing circulating cholesterol droplets, and impairing HDL maturation [157]. Therefore, augmented renal proteinuria caused profound alteration in plasma and liver enzymes function, avoiding HDL maturation, determining profound impact in several organs including kidneys [157].

It is clear that kidney injury could alter HDL levels and composition, but further studies are needed to evaluate the effects of other receptors, as SR-BI and ABACG1 in renal HDL catabolism or transcytosis.

## 6. SARS-CoV-2: Mechanism of Cellular Infection

The epidemic of respiratory disease caused by SARS-Coronavirus-2 (SARS-CoV-2) that first emerged in Wuhan and then spread worldwide, causing more than 3 million deaths and economic fatalities, forced researchers to dissect the molecular mechanisms of cellular infection [158].

According to the phylogenetic profile, the Coronaviridae family comprises viruses with genetic heterogeneity that permit differentiation in four genera: α-coronavirus, β-coronavirus, γ-coronavirus, and δ-coronavirus. SARS-CoV-2 is a β-coronavirus belonging to the subgenus botulinum of Coronaviridae with a single-strand positive-sense RNA genome [159]. The amount of genetic viral code is unusually large, containing from 26 to 32 kilobases with an extremely high degree of variability [160]. The sequencing of SARS-CoV-2 revealed 96.2% identity with a bat coronavirus (BatCoV RaTG13). In addition, SARS-CoV-2 shares 79.6% homology with SARS-CoV, also derived from bats, and palm civet and 51.8% identity with MERS-CoV [161].

It is commonly accepted that SARS-COV-2 targets airways and may severely involve lung; however, SARS-CoV-2 emerges to be detected in multiple organs, including the heart, liver, brain, and more importantly, the kidneys [162]. The favored SARS-CoV-2 tropism for the respiratory system is allowed by the attachment to angiotensin-converting enzyme 2 (ACE-2). ACE-2 is a membrane-anchored carboxypeptidase commonly expressed by airway epithelial and type I and II alveolar epithelial cells as well as in organs that regulate blood pressure as myocardial cells, kidney proximal tubule cells, and podocytes. Finally, ACE-2 is also present in bladder urothelial cells, oesophageal epithelial cells, enterocytes, and cholangiocytes [163]. As a consequence of this heterogeneity in the infection site, COVID-19 syndrome widely varies from a uncomplicated weakness, fever, dry cough to severe pneumonia associated with acute respiratory distress syndrome, multi-organ failure, sepsis including kidney failure, cardiac injury, and thrombosis [164].

From a pathophysiology perspective, the binding of SARS-CoV-2 to human ACE-2 on host cells occurs by viral spike proteins (S) that are formed by two major subunits. Firstly, the S1 subunit is necessary for the attachment of the host cell receptor, whereas S2 is important for the subsequent viral particle penetration [165]. Immediately after recognition, the large ectodomain of the spike protein becomes available as substrate for several host peptidases as furin-like protease, Transmembrane Protease Serine 2 (TMPRSS2) serine protease which cut at the border of domains S1 and S2 (cleavage site S1/S2) and at the S2’ site. This process induces conformational changes of the S2 site that facilitate the fusion of the viral and host cellular membrane. It should be specified that the mechanism of S1/S2 action is strictly pH dependent and includes different enzymes at various stages of the viral life cycle. In particular, cleavage occurs at the level of the cell membrane, as well as in endosomes, or at the level of the endoplasmic reticulum and Golgi apparatus in the phase of protein biosynthesis. After entry into the cytoplasm, the viral genome encodes the cysteine papain-like peptidases (PLPs) and chymotrypsin-like cysteine 3C-like peptidase (3CL^Pro^), also known as main peptidase (M^Pro^), which are necessary for mediating viral genome transcription and replication. The viral RNA genome is translated into 2 large polyproteins, pp1a and pp1ab, which are subsequently cleaved into different non-structural proteins (Nsps), building a replicase–transcriptase complex that is crucial for viral transcription, replication, and spread [166].

Recent studies shed new insight in the exact interaction of C-terminal domain (also called the receptor-binding domain [RBD]) of S1 to the NH-terminal site of human ACE. More specifically, by elegant studies, Shang et al. demonstrated that the affinity of SARS-CoV 2 for ACE-2 domain is higher than for other coronavirus (i.e., SARS-CoV), RBD can switch from a standing up position (for active infection) to a lying down position (to be less recognizable by immune surveillance), and lastly, SARS-CoV2 needs to be proteolytically activated by surface protease as TMPRSS2 and lysosomial cathepsin [165].

In conclusion, all these recently discovered processes add new layers of complexity in the pathophysiological mechanisms of SARS-CoV2 infection, arising the urgent need of combinatory inhibition strategies to counteract the viral infectivity, immune evasion, and widespread into multiorgan host cells.

## 7. SARS-CoV-2 Exploits the SR-BI Associated HDL to Promote its Entry

Despite the great efforts to elucidate the molecular mechanisms of SARS-CoV2 cell entry, the pathogenetic mechanisms have not been fully clarified and there are no effective therapies to stop or reverse the occurred infection. The partial inhibition obtained by ACE-2 antibodies in contraposition to maximum neutralization efficacy of monoclonal antibodies targeting the SARS-2-S1 N-terminal domain suggested that viral particles exploited additional receptors and channels for host cell entry [167,168,169]. In accordance, besides ACE-2, other receptors, such as Dipeptidyl peptidase 4 (DPP4) or aminopeptidase N, have been proposed to play a role in SARS-CoV2 entry in target cells. Specifically, DPP4, also known as cluster of differentiation 26 (CD26), a serine exopeptidase expressed ubiquitously in lung, kidney, liver, gut, and immune cells, has been investigated to allow virus SARS-CoV-2 cell adhesion/virulence [170].

Among additional mechanisms of enhancement SARS-CoV-2 entry, SR-BI has been recently examined to play a central role in augmenting virus attachment. As previously underlined, SR-BI is a kind of membrane protein with a molecular weight of 82 kDa, that acts as the major HDL scavenger receptor and mediates the selective HDL-cholesterol uptake of cells [171]. This cholesterol delivery system is well studied in hepatocytes as well as steroidogenic cells as adipocytes, adrenal cells but also in fibroblasts, macrophages, ovarian cells, and testicular Leydig cells [172]. Notably, epithelial alveolar type II cells also express SR-BI, in this location, SR-BI is involved in vitamin E intake, specially from HDL [173,174].

In an interesting in vitro study, Wei et al. demonstrated that SARS-COV2 exploited the physiological function of SR-BI to mediate cholesterol-bound HDL intake to promote its cellular entry [169]. In other words, even if SR-BI cannot bind to the SARS-2-S protein directly, the receptor acts as a linker molecule that recruits viral particles to come into proximity with ACE-2 by association with HDL. In accordance, the SR-BI expression conferred the greatest cell susceptibility to SARS-CoV-2 only when co-expressed with ACE-2. Therefore, it emerged that treatment of cultured cells with pharmacological SR-BI antagonists, could inhibit HDL-enhanced SARS-CoV-2 infection inhibition. Furthermore, also blockade of the cholesterol-binding site on SARS-2-S1 with a monoclonal antibody showed similar effects. In line, ITX 5601, a clinically approved inhibitor of HCV infection, strongly inhibits SARS-CoV-2 infection of cultured cells. Strikingly, besides lung, authors further showed that SR-BI is co-expressed with ACE2, predominately in lower respiratory tissues that are more affected by COVID-19 and in several extrapulmonary tissues as retina, testis, as well as kidney which could indicate an extended trophism for extrapulmonary tissues, thereby to the multiple-organ pathologies of COVID-19 [175].

SARS-COV-2 has a lipid envelope that merges with the host cell through endocytosis, internalizing its components in the cell. Recently, cell experiments showed that the susceptibility of the virus to fusion with the host cell membrane augmented when cholesterol was added to medium culture [176]. For example, cholesterol-supplemented mouse fibroblasts showed increased susceptibility to fusion with murine hepatitis virus [177]. ACE-2 resides mainly within lipid rafts, furthermore, in lipid raft areas, there are also caveolins, clathrins, and dynamin, that are molecules with a central role in the incorporation of viruses [178]. Similarly, uptake mechanisms dependent from these molecules and on the presence of lipid rafts have been also identified for the simian virus (SV40) [176].

In the context of HDL and lipoproteins metabolism, it could be interesting to note the existence of an association between ACE insertion/deletion polymorphism with HDL level and cardiovascular disease risk factors. As recently demonstrated, an ACE polymorphism in men was significantly associated with reduced HDL cholesterol levels [179]. Cholesterol is an essential lipid component of vertebrate cell membranes, mainly through lipid rafts, i.e., microdomains enriched in cholesterol and sphingolipids. Lipid rafts have been suggested to play a fundamental role in several biological processes such as signal transduction, membrane trafficking, cytoskeletal organization [180,181].

Given their unique protein composition, lipid raft microdomains are essential for the endocytosis-mediated process and serve as a platform and docking site for viruses to enter the host cell [178,180]. Emerging evidence revealed that cholesterol diminution from cellular membranes has been shown to hamper SARS-CoV-2 infection [182]. This finding led several researchers to speculate that SARS-CoV-2 may exploit the physiological function of SR-BI to achieve its entry and fusion processes [183].

Furthermore, Meher et al. reported the effect of membrane cholesterol on the structure of the fusion peptide (residues 770–788) of S2 glycoprotein of SARS-CoV. Authors elegantly demonstrated that S2 binding affinity augmented with increasing levels of membrane cholesterol [184]. On the other hand, cholesterol decline disturbs the virion membrane. Through the interference with lipid-dependent attachment to human host cells, sterols and cyclodextins can reduce the infectivity of CoVs. [185]. In vitro depletion of membrane-bound cholesterol in ACE2-expressing cells led to a reduced infectivity of CoVs, since the binding of the spike protein was reduced by half. The mechanism of employing lipid raft rich in cholesterol together with the utilization of another type of lipid called monosialotetrahexosylganglioside 1 (GM1), to enter mammalian cells in culture is shared by both SARS-CoV and SARS-CoV-2. This concept has emerged by the reduction of infection in cells treated with a compound called methyl-β-cyclodextrin (MβCD) able to deplete cholesterol [186]. The cholesterol removal by MβCD significantly dissociate the number of bonds between ACE-2 membrane protein and viral S glycoproteins [187].

Some studies showed that MβCD treatment dose-dependently reduced expression of ACE-2 in the cell membrane, also reducing the infectivity of SARS-CoV2 [177]. The lipophilic core permits the contact of these molecules with lipid rafts. These harmless macromolecules are able to mimic binding domains for the enveloped virus, competing with host cell attack sites, thereby reducing infection.

Additionally, interaction of phytosterols with lipid raft molecules can lead to a decrease of membrane cholesterol content or destabilization of its structure, thereby affecting viral infectivity [177]. In addition, the viral infectivity is modulated by homeostatic control of cholesterol content and fatty acid metabolism [188]. More recently, Henrich, S. E. et al. elegantly demonstrated that SARS-CoV-2 viral entry is impaired by SR-BI genetic knockdown, suggesting that SR-BI is a co-receptor for SARS-CoV-2. Interestingly, authors also demonstrated that inorganic core nanoparticles around which HDL-associated protein (apolipoprotein A-I) and lipids were clustered (HDL-particles) targeted SR-BI to inhibit SARS-CoV-2 entry. Indeed, the superficial resemblance to HDLs caused these nanoparticles to firmly bind to SR-BI. These nanoparticles are characterized by the ability to strongly inhibit the entry of exosomes, which are extracellular lipid vesicles. Finally, by altering the type of lipids assembled on the surface of these nanoparticles, they could target Gram-negative bacterial LPS and prevent the LPS-mediated release of potent pro-inflammatory signalling.

Based on these findings, HDL nanoparticles could offer a promising strategy to prevent infection with SARS-CoV-2. Strikingly, they could also be investigated as a possible treatment for other cholesterol-dependent viral infections that are also based on lipid rafts for their successful entry into host cells [186].

## 8. Dysregulation of Lipidomic Profile during SARS-CoV-2 Infection

Numerous observational and meta-analyses studies have shown that hypertension, cardiovascular disease, and diabetes mellitus can predispose to higher severity and mortality of COVID-19 [189,190]. However, very few studies have been performed on dyslipidemia [182,191]. Some years before the occurrence of COVID-19 pandemic, the role of lipid metabolism in coronavirus disease was already investigated by several groups [192]. However, the relationship between cholesterol and SARS-CoV-2 viral infection and COVID-19 syndrome is still poorly understood.

Obesity and impaired metabolic health might be strongly associated with increased risk of severe COVID-19 [191]. In accordance, it is well known that HDL-mediated cholesterol efflux and selective cholesterol transport are hallmarks of many chronic metabolic diseases.

Besides pneumonia, COVID-19 patients develop abnormalities, such as lymphocytopenia, progressive increase in pro-inflammatory cytokine levels (the cytokine storm) and C-reactive protein (CRP), as well as a decrease in total protein, albumin, ApoA-I, HDL-cholesterol, and triglycerides, together with lowered CD3^+^T, CD8^+^T cell level [193]. As already discussed, HDL-cholesterol and ApoA-I play protective roles in the maintenance of health and have beneficial effects on the lungs and other affected organs.

In the context of viral infections, several studies reported a substantial alteration in lipid metabolism and lipoprotein composition [194]. Looking inside the mechanisms of infection, lipids are crucial in the infection process, as they are important structural components of cellular and subcellular organellar membranes [195]. Viral incorporation requires the binding of the virus to the host cell membrane, activating an endocytosis mechanism. As previously underlined, the membrane lipid composition, and particularly the lipid rafts, influence this process [178]. When inside the cell, the virus replicates exploiting the metabolic machinery of the infected cell. The newly synthesized viral particles exit the cells again by crossing the lipid-rich cell membrane. From this perspective, it could appear clearer why infectious diseases as HIV and HCV are associated with low HDL cholesterol (HDL-C) concentrations and sometimes with low LDL cholesterol (LDL-C) concentrations [196,197,198]. In line with this hypothesis, also during the SARS-CoV-2 infection, low LDL-C, HDL-C, and triglyceride (TG) levels have been defined to be associated with an amplified infection severity.

In the last months, a plethora of clinical trials (e.g., NCT04384705), observational and prospective studies, and a large series of case reports led to the central conclusion that declined serum HDL-cholesterol is a predictive factor for COVID-19 severity infection and poor outcome [199,200,201]. In a retrospective study, Huang W. et al. analyzed circulating level of HDL-C and LDL-C level in a cohort of 2623 COVID-19 patients divided into three groups—non-critically ill, critically ill, and death groups [201]. Authors found that LDL and HDL levels were significantly lower in critically ill and death groups compared to non-critically ill groups [201]. Furthermore, low HDL-C appeared to be an independent risk factor for developing severe events, which recommended that COVID-19 patients with low HDL-C needed more intensive treatment [194]. In a recent study, Hu et al. analyzed the lipid profile and other clinical features of COVID-19 patients. Authors found that serum triglycerides, HDL-, and LDL-cholesterol were significantly lower in COVID-19 patients [183,193]. More specifically, the alterations in serum lipidomic appeared to be gender dependent, with male patients demonstrating significantly lower HDL-cholesterol and a higher number of monocytes and a higher lactate dehydrogenase compared to female patients [193].

A significant decrease in the level of HDL-cholesterol was observed only in critical cases of COVID-19, while significant decrease of TC and LDL-cholesterol was observed in all patient groups (mild, severe, critical). Accordingly, it seems that hypolipidemia occurs in patients with mild symptoms and escalates with the progression and severity of the disease. Despite all these emerging data, no interventional studies have been performed to provide strong evidence that infection with SARS-CoV-2 is modified by HDL level. For a mechanistical point of view, considering that the alteration in the lipidomic profile was associated with increased serum levels of alanine aminotransferase (ALT), alkaline phosphatase (ALP) and aspartate aminotransferase (AST), researchers hypothesized liver damage was as a consequence of the SARS-CoV-2 infection. Alternatively, HDL and LDL particles levels may also be affected by increase in IL-6 levels [202]. In contrast, another study observed a significantly increased level of serum LDL compared to the reference population where levels of HDL-cholesterol and TC were inversely correlated with the severity of COVID-19 [199].

**Figure 3:**
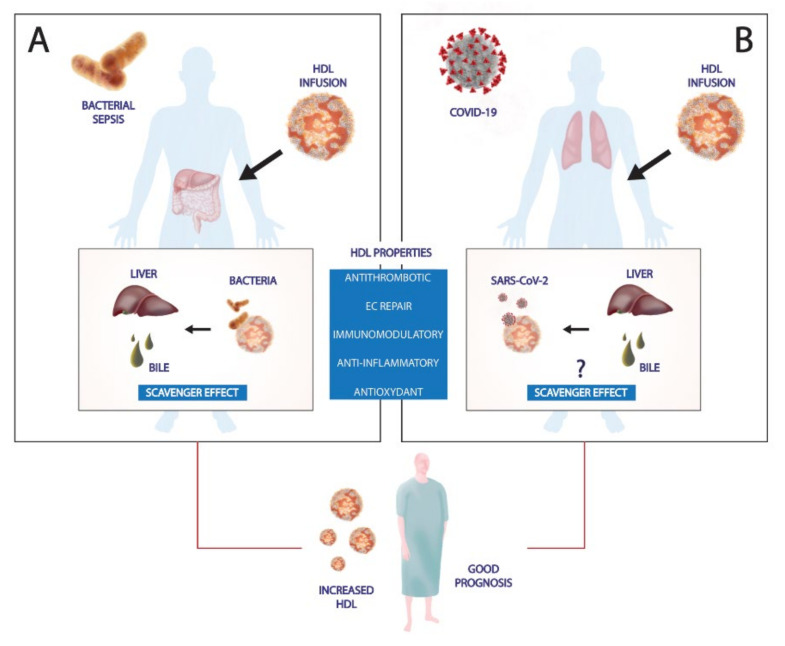
Role of HDLs in counteracting bacterial sepsis and SARS-CoV2 infection. (**A**) HDLs exert scavenger effects in bacterial infections. HDLs are able to bind and neutralize Gram-positive and Gram-negative components and promote their clearance via the liver and subsequent bile elimination. In addition, HDL has multiple protective functions. Preclinical studies demonstrated that exogenous HDL injection increased survival rate, reducing systemic inflammatory response. (**B**) Recent studies support the notion that SARS-CoV-2 has a specific affinity for cholesterol and HDL. However, it is not clear if HDL could interfere with SARS-CoV-2 entry into host cells. Serum lipid profile changes and reduced HDL levels are correlated to severe forms. Therefore, HDL-based therapies or drugs that modulate cholesterol metabolism could increase HDL levels, provide pleiotropic functions that ameliorate clinical outcome.

Furthermore, a role for these lipids in immune and inflammatory mechanisms has been demonstrated. As previously discussed, besides their major function in RCT, HDL particles play an essential role in immunological surveillance and in the modulation of infectious diseases (Figure 3).

Like sepsis disease, the overwhelmed inflammatory response and the cytokine storm are the principal mechanisms in COVID-19 progression and severity. It is well recognized that inflammation alters hepatic apolipoprotein gene expression. Furthermore, the increased pro-inflammatory SAA, with an higher affinity for HDL and displacing activity can decrease ApoA-I levels in HDL [203]. Moreover, in the setting of acute inflammation, decreased plasma levels of LCAT have been demonstrated to alter HDL function and further deteriorate the inflammatory response [204]. In COVID-19 patients, in line with these observations, SAA plasma levels are elevated and correlated with disease severity [205]. Finally, systemic inflammation as observed during cytokine storm during COVID-19 can inactivate PON1, the antioxidant enzyme present in HDL which further compromises HDL function [206]. Indeed, decreased function of PON1 correlated with poor prognosis in cardiovascular disease and was detected to be diminished in both inflammatory and infectious contexts [206,207].

Different HDL-based therapeutic options have been suggested for the management of SARS-CoV-2 pro-inflammatory and immunological complication. For instance, HDL functionality could be improved by increasing LCAT activity or by replacement with other apolipoproteins, such as ApoA-I mimetic peptides.

## 9. Similarities Sepsis and CRS-SARS-Cov-2 Infection: Focus on Coagulation and Complement Activation

The clinical manifestation of COVID-19 syndrome starting with mild symptoms as dry cough, nasal congestion and evolving into severe ARDS, systemic inflammation, and progression to organ dysfunction share several pathophysiological and clinical features with bacterial sepsis [208]. In line, critical cases of SARS-COV-2 patients suffer from respiratory failure, multiple organ failure until to septic shock [19]. Particularly, both sepsis and COVID-19 patients experience high cytokine production, leukopenia, hypotension, consumptive thrombocytopenia, haemolytic anaemia, vascular microthrombosis, disseminated intravascular coagulation (DIC) [19,208]. From a renal perspective, SARS-COV2 and sepsis patients exhibited reduced GFR [209]. Other similarities between sepsis and COVID-19 are detectable at the liver level, as assessed by increased bilirubin and hypoalbuminemia, which are the consequence of critical hepatotoxicity and strongly decreased protein synthesis in parenchymal cells [210]. Interestingly, coronary heart disease and dementia are common in both COVID-19 and sepsis patients [208,211,212].

Taking into account the manifest parallelism between the immunopathogenesis and clinical manifestations of sepsis and COVID-19, it has been supposed that understanding sepsis mechanisms could provide more elucidation on COVID-19 pathophysiology and management [208,213].

A special focus in the similarities between sepsis and COVID-19 syndrome should be reserved for abnormalities into coagulation system. Increased disseminated intravascular coagulation and vascular microthrombosis have been observed both in septic and COVID-19 patients. Hypercoagulability is a derangement of hemostasis characterized by sharp D-dimer. It has been suggested that SARS-CoV-2 attacks vascular endothelial cells, promoting the endothelial dysfunction and leading to abnormal coagulation [208]. However, the haematological features of COVID-19-induced coagulopathy is quite different from that in typical sepsis. From a side, sepsis is characterized by systemic hypercoagulation and impaired fibrinolysis, whereas severe COVID-19-induced coagulopathy promotes local thrombus formation [208,214]. Additionally, venous thromboembolism and arterial thrombosis are often observed in coagulopathy that characterizes COVID-19 patients compared to non-COVID-19 induced coagulopathy. Moreover, COVID-19 complications include not only cytokine storm but also an acute respiratory failure, both the conditions are capable of damaging distant organs [208,215].

Several potential mechanisms could explain the coagulation impairment caused by COVID19. The major mechanism that has widely been described is the “cytokine storm syndrome” [216]. More precisely, SARS-CoV-2 rapidly activates Th1 cells to secrete proinflammatory cytokines, such as granulocyte-macrophage colony-stimulating factor (GM-CSF) and IL-6. GM-CSF further triggers CD14+CD16+ inflammatory monocytes to produce large quantities of IL-6, TNF-α, and other cytokines. The cytokine storm promotes the further infiltration of macrophages and neutrophils into the lung tissue, with consistent damage. In comparison with sepsis, also membrane-bound immune receptors (i.e., TLRs) may contribute to an imbalanced inflammatory response and weak IFN-γ induction [109,113,208,217]. The cytokine storm in COVID-19 is characterized by a high expression of IL-6 and TNF-α. These soluble mediators are typically assessed at high levels in patients with sepsis associated with hypercoagulable condition, as observed in disseminated intravascular coagulation. However, IL-6 has been described also as an essential mediator in initiating hypercoagulation [218], through activation of TF. Patients with COVID-19 showed an increase in TF expression as a possible consequence of lungs tissue damage and inflammation, leading to induction of IL-6. Lastly, fibrinogen and factor VIII production are also fueled by IL-6, which is particularly detrimental at the endothelial level. IL-6 has been shown to induce vascular permeability by stimulating vascular endothelial growth factor (VEGF) secretion [219].

In addition, the binding to ACE-2 is followed by an increase in Ang II, whicht can activate the pro-inflammatory NF-κB pathway and induce TNF-α and the soluble form of IL-6Ra (sIL-6Ra) via disintegrin and metalloprotease 17 (ADAM17) [220]. IL-6 binds to sIL-6R through gp130 to form the IL-6-sIL-6R complex, which can activate signal transducer and activator of transcription 3 (STAT3) in non-immune cells. Both NF-κB and STAT3 are capable of activating the IL-6 amplifier to induce various proinflammatory cytokines and chemokines, including vascular endothelial growth factor, MCP-1, IL-8, and IL-6 [220]. In parallelism with sepsis, the NF-κB pathway activation is a central signaling activated after Pathogen Recognition Receptors (PRR) recognition by monocytes and neutrophils, during Gram-negative infection.

Aberrant complement system activation represents another central mechanism common to sepsis and SARS-COV2 syndrome. Primarily, extrinsic and intrinsic coagulation pathways are linked with complement factors to maintain homeostasis. During systemic inflammation, complement anaphylatoxins C3a and C5a have been associated with strong upregulation of coagulation factors, leading to disseminated intravascular coagulation [221,222]. In the context of infections, complement activation-induced pro-coagulation through the actions of C5a which activates TF, mannan binding lectin serine protease (MASP)-1 which cleaves fibrinogen and factor XIII, and therefore activates coagulation. In line with this observation, post-mortem studies of COVID-19 patients have shown increased interalveolar endothelial deposits of MBL, MASP2, C4b, C3b, and C5b-9, as well as C5b-9 deposits in the glomeruli of kidneys [221].

In a recent proteomics analysis of sera from COVID-19 patients, D’Alessandro A. et al. stratified patients by circulating levels of IL-6 and correlated to markers of inflammation and renal function. In patients with high IL-6 level, authors identified significant dysregulation in serum levels of various coagulation factors, accompanied by increased levels of antifibrinolytic components, including several serine protease inhibitors (SERPINs). These changes were accompanied by the increase of the complement activation which is consistent with an exacerbation of the acute phase response. This clear augment in the levels of inhibitory components of the fibrinolytic cascade in severe COVID-19 disease, still confirmed the differences between SARS-COV2 and sepsis [223].

Finally, in order to evaluate whether there are differences in the immune system status of critically ill COVID-19 and sepsis, Dong X. et al. analyzed in a total of 107 patients, neutrophil, lymphocyte, and monocyte counts, infection biomarkers (C-reactive protein, ferritin, and procalcitonin levels), lymphocyte subset counts (i.e., CD4^+^, CD8^+^, B cell, and NK cell) [213]. Authors found that the immunological profile was similar in bacterial sepsis patients and SARS-CoV-2 sepsis patients; however, the cytokine storm was milder, while immunoglobulin and complement protein levels were typically higher in SARS-CoV-2 patients.

In brief, there are both similarities and discrepancies in the immune system and coagulation status of sepsis and SARS-CoV-2. Whatever the mechanisms in sepsis and COVID-19, dyslipidemia has a close relationship with the immune system and coagulation. Therefore, a better knowledge of lipid metabolism in systemic inflammatory diseases could provide new therapeutic targets.

## 10. Renal Implication in COVID-19 Disease

Although lungs are the primary target of COVID-19, people severely affected present extrapulmonary co-morbidities that lead to multiorgan failure [224]. The occurrence of AKI as defined by KDIGO criteria in COVID-19 is 36% [225,226]. When considering possible subclinical kidney damage, the prevalence might be higher and up to 41% of COVID-19 patients with urine sediment positive for red blood cells. A recent prospective study of 701 mild to moderate patients showed that 43.9% of them presented proteinuria, 26.7% hematuria, and only 13% had altered renal function [227]. In most serious cases, renal damage is severe enough to require dialysis and the incidence of mortality is highly increased [209,228]. Moreover, patients that discharged after COVID-19-associated AKI showed a median creatinine of 1.7 mg/dL, consistent with persistent kidney damage. Recently, a clinical research report showed worse renal function in survivors after hospital discharge. The lower recovery rate has been recorded in one third of 3993 hospitalized patients and it is due to the severity of AKI in these patients [229].

The effect of COVID-19 on the kidneys is not completely clear. The pathogenesis of SARS-CoV-2–induced AKI is multifactorial and involved several mechanisms, such as the local disruption of RAAS homeostasis, the hemodynamic instability, the cytokine storm, and direct viral cytopathic damage through ACE-2 [226,230].

The function of ACE-2 in normal human physiology is to regulate blood pressure via inhibition of the angiotensin–renin–aldosterone pathways. As previously described, ACE-2 has a wide distribution in multiple organs, including the nose, lungs, kidneys, liver, blood vessels, immune system, and the brain. During early SARS-CoV-2 infection and viral spread within body tissues, the conversion of angiotensin II to angiotensin by ACE2 is impaired. Higher levels of angiotensin II are associated with vasoconstriction, kidney failure, heart disease, apoptosis, and oxidative processes that accelerate disease progression [231]. Renal ACE-2 expression is fundamental in the pathogenesis of kidney failure in several diseases [232]. Increasing evidence have highlighted that ACE-2 impairment is implicated in both acute and chronic renal diseases and determines the loss of RAS homeostasis and parenchymal pathological changes [232,233]. The higher tropism of SARS-CoV-2 for renal parenchyma is probably due to ACE2 affinity for viral spike protein S1, which could allow virus entry, its replication and direct damage to surrounding tissue. In addition, in silico analysis performed on data sets of single-cell RNA sequencing revealed that RNA of genes that are considered to facilitate SARS-CoV-2 infection (i.e., RNA of ACE-2, transmembrane serine protease 2, and cathepsin L) are predominantly enriched in kidney cells. This enrichment suggested an increased susceptibility of renal cells to SARS-CoV-2 infection [234,235].

Histological analysis of renal biopsies of COVID-19 patients revealed the presence of viral nucleoprotein (NP) antigens in the cytoplasm of renal tubular epithelial cells, suggesting a direct viral infection [236]. Indeed, acute tubular necrosis (ATN) is the most common pathological change observed in COVID-19 patients with AKI [234,237]. Viral endocytosis could be mediated by ACE-2 receptor internalization with the involvement of specific proteases, including cysteine protease cathepsin B/L, glutamyl aminopeptidase, and serine protease DPP4, which might facilitate SARS-CoV-2 binding to ACE-2 [238]. Interestingly, recent evidence showed that SARS-CoV-2 could infect host cells through the binding to another transmembrane receptor known as CD147, which is highly expressed in endothelial cells and it was also found in renal parenchyma [239]. Recently, Werion et al. reported that kidneys from patients with COVID-19 showed prominent tubular injury, with brush border loss, acute tubular necrosis, intraluminal debris, and a marked decrease of surface Megalin expression; electron microscopy showed viral particles resembling in vacuoles of the endoplasmic reticulum of proximal tubule cells [240]. The loss of Megalin protein on the brush border of tubular cells could impair renal HDL catabolism, that could probably influence plasma concentration of HDL and ApoA-I in COVID-19 patients [241].

Su et al. demonstrated the involvement of podocytes in COVID-19 disease, showing the presence of viral antigens in their cytoplasm [234]. Therefore, it is reasonable that SARS-CoV-2 firstly invades podocytes and then renal proximal tubular cells. The cytopathic effects of the virus induces podocyte vacuolation, foot process effacement, and cell detachment with consequent loss of integrity and functionality of the glomerular filtration barrier [234]. These pathological changes could determine nephron hyperfiltration and increased proteinuria [242]. In addition, tubular damage could also augment proteinuria, usually with a mild intensity [243].

Cytokines play a pivotal role in determining organ dysfunction during SARS-CoV-2 infection. Although innate immune response represents the first line of defense against viral infections, when it is excessive and dysregulated induces multi-organ damage [244]. Indeed, serum cytokines (IL-6, IL-1B, IFN-gamma, IP-10, and MCP-1) and chemokines (CCL2, CCL-5, CCL3, IP 10) are significantly increased during SARS-CoV-2 infection and they correlate with disease severity and worsen clinical outcome [245,246]. In addition, IL-6 is considered a good biomarker for early diagnosis and prediction of clinical outcome and AKI development [247].

Finally, a hypercoagulopathy state has been widely reported as a potential mechanism involved in the severity of disease [248]. The hematological profile of COVID-19 patients showed high plasma levels of reactive protein C, fibrinogen, D-dimer, and ferritin, which are correlated with thrombocytopenia [249]. Several studies reported evidence of disseminated intravascular coagulation, with signs of thrombotic microangiopathy in different organs, including the kidneys [250]. In addition, the activation of complement system, the endothelial dysfunction, the increased levels of cytokines, and the involvement of monocytes and macrophages predispose to the activation of coagulation cascade [249]. In particular, the role of the complement system in COVID-19-associated coagulopathy is supported by numerous data that demonstrate MAC activation in the microvasculature of many organs and in particular the kidneys [251]. In addition, the presence of anticardiolipin-like antibody may induce neutrophils aggregation, leading to thrombotic events [252]. In addition, the loss of RAAS homeostasis due to reduced ACE-2 activity could promote hypercoagulability [253].

The incidence of AKI in COVID-19 disease is associated with demographic parameters, clinical conditions at hospital admission and specific genetic traits [254,255]. Recent evidence showed that patients with advanced age were more susceptible to AKI occurrence during SARS-CoV-2 infection, probably due to physiological renal aging. Moreover, some genetic polymorphism could affect kidney function predisposing to organ damage. Several studies have reported higher frequency of collapsing glomerulopathy in African American patients with COVID-19 [256]. The mechanisms of collapsing glomerulopathy and podocytopathy are still under investigation. It is plausible that genetic polymorphisms of the Apolipoprotein L1 (APOL1) allele, which is more common in this ethnic group, could confer higher susceptibility to develop renal damage during SARS-CoV-2 infection [256].

As previously underlined, hypolipidemia occurs in patients with mild symptoms and escalates with the progression of the disease and multi-organ damage. Considering the multi-protective roles of HDL in preventing systemic inflammatory response, endothelial dysfunction, coagulation cascade, and renal damage in sepsis disease, HDL replacement therapy could offer a new strategic treatment to ameliorate kidney function in this context.

## 11. HDL-Based Therapies in Preclinical Studies

Benefits of recombinant HDL and HDL mimetic peptides have been widely demonstrated in several preclinical models of endotoxemia and sepsis and in endotoxemia human studies. The first evidence was derived from the intraperitoneal (IP) administration of naked ApoA-I in endotoxemic rats. The authors observed that administration of ApoA-I after 1 h post infection significantly reduced cytokines levels and increased 5-day survival rate [257]. Accordingly, the intravenous infusion of ApoA-I at an increased dose (100 mg/kg), 1 h post-LPS challenge, increased 3-day survival rate and average survival time compared to controls [258]. In addition, adenovirus-mediated ApoA-I overexpression in a mouse model showed protective effects against LPS-induced systemic inflammation and multiple organ damage [259]. The protective effects of HDL and ApoA-I were also observed in a mouse model of LPS-induced inflammation, in which the impact of ApoA-I gene transfer determined a strong decrease of TLR-4 expression and activation, reducing endothelial dysfunction, inflammatory response, and lung damage [260].

CSL-111, a recombinandt HDL (rHDL), although originally produced for atherosclerosis treatment [261], has shown efficacy in reducing the inflammatory response during LPS-induced endotoxemia in an in vitro system and in rabbit [262] and human models [263,264].

In a clinical double-blind, randomized, placebo-controlled, cross-over study, the infusion of CSL-111 was shown to decrease the procoagulant state caused by endotoxin exposure, reduce monocyte activation and cytokine production (i.e., TNF-α, IL-6, and IL-8), ameliorating hemodynamic parameters and clinical symptoms [263,264].

ApoA-I Milano, a natural variant of ApoA-I, has been widely studied in the context of cardiovascular disease (CVD) in a Phase I trial [262] and other further clinical studies. Recently, Zhang et al. demonstrated that ApoA-I was also efficacious against inflammation in endotoxemic rat model [265]. Recently, Tanaka et al. reported that CSL-111 infusion improved survival in different experimental mouse models of sepsis (CLP or intraperitoneal injection of Escherichia coli or Pseudomonas aeruginosa), reducing inflammation in both plasma and organs and decreasing bacterial count [266].

Several ApoA-I mimetic peptides have been synthetized and studied in the last years. They are structurally similar to the native ApoA-I and are able to bind phospholipids and reproduce the same functions of native HDL in terms of cholesterol efflux, interaction with specific receptors and enzymes as LCAT and PON, and bacterial detoxification [267,268]. Eighteen amino acid peptide 18A has been the first HDL mimetic peptide investigated in this setting and its use demonstrated a significant increase in survival rate in septic mice [269].

Among HDL mimetic peptides, L-4F has been more widely employed in several preclinical model of sepsis and has been shown to block production of cytokines, reverse sepsis-induced hypotension, prevent organ damage, and restore renal, hepatic, and cardiac function, and increase survival rate [17,51,270].

Other experimental studies were reported and summarized in Table 1.

## 12. Conclusions

The cytokine storm underlying systemic inflammatory diseases induced an immune-mediated inflammatory dyslipidemia, typically characterized by low and dysfunctional HDL levels [206]. These abnormalities might be modified using pharmacological agents that increase circulating HDL and ApoA-I. Experimental and clinical investigations, including Phase 2 clinical trials for treatment of cardiovascular disease, have demonstrated that the infusion of HDL improves endothelial function and reduces oxidative stress, inflammation, and platelet aggregation [64,241,276,277,278,279,280,281]. Among the different mechanism of actions of rHDL and HDL-mimetic complexes, the well documented capacity of those lipoproteins to correct the dyslipidemia and inhibit the induction of the inflammation cascade, could be the primum movens to reduce systemic inflammation. Further efforts are required to understand the role of HDL dysfunction in this setting and to design clinical studies to provide access to HDL-based therapies for systemic inflammatory diseases with severe cytokine release syndrome and renal injury and limited treatment alternatives.

## Figures and Tables

**Table 1:** HDL-based therapies in preclinical studies.

References	Study Protocol	Main Results
Imai et al., 2003 [257]	Wistar rats. IP infusion of 10 mg/kg 1 h post-LPS infusion (1 mg/kg for TNF analysis, 5 mg/kg for survival analysis	ApoA-I inhibited the release of serum TNF-Alpha and improved the survival rates from 0 to 90%
Yan et al., 2006 [258]	Balb/c mice. IV infusion of 100 mg/kg 1 h post LPS infusion (5 mg/kg)	ApoA-I could significantly inhibit LPS-induced increases in L-1beta and TNF-α levels in serum (*p* < 0.05, respectively), as well as in the IL-1β, TNF-α, and IL-6 levels in BAL fluid. ApoA-I significantly decreased the mortality and increased the survival time.
Van Linthout et al. [260]	8-week-old male C57BL/6 mice. IV injection of particles containing the E1E3E4-deleted adenoviral vector Ad.hapoA–I, expressing human ApoA–I (5 × 10^10^)	Apo A-I gene transfer mice were protected from LPS effects and presented decreased levels of lung TLR4 and lung MyD88 mRNA expression. Neutrophil infiltration, lung edema, and mortality were significantly attenuated following ApoA–I transfer. In vitro, supplementation of HDL or ApoA–I to human microvascular endothelial cells 24 h before LPS stimulation reduced TLR4 expression and endothelial dysfucntion.
Zhang et al., 2015 [265]	Wistar rats. Prophylactic IV infusion of rHDL 40 mg/kg	Suppressed proinflammatory cytokines and adhesion molecule increase in TNF-α, IL1β, IL-6, and intercellular adhesion molecule 1. rHDL pretreatment inhibited lipid peroxidation and enhanced total antioxidant capacity in vivo.
Casas et al., 1995 [262]	Rabbits. Prophylactic IV infusion of CSL-111 25–50 mg/kg	Reduced TNF-α for both rHDLdoses Reduced hypotension with rHDL 50 mg/kg
Hubsch et al., 1995 [271]	Rabbits - Prophylactic IV infusion 75 mg/kg - IV infusion after 1 h post-bacterial challenge	-Prophylactic rHDL: reduced metabolic acidosis, TNF-α - Treatment: reduced LPS, metabolic acidosis, improved renal function, no significant effects on hypotension and TNF-α
Zhang et al., 2009 [17]	Sprague-Dawley rats. IP infusion of 4F peptide 10 mg/kg 6 h post-CLP	Reduced IL-6. Improved cardiac output and plasma volume. Improved survival rate.
Dai et al., 2010 [51]	Sprague-Dawley rats. 4F peptide 10 mg/kg post-LPS (10 or 30 mg/kg)	Reduced hypotension at 6 h; increased HDL plasma levels; improved survival rate at 24 h
Moreira et al., 2014 [270]	Wistar rats. IP infusion of 4F peptide 10 mg/kg 6 h post-CLP	Restored expression levels of Slit2, Robo4, eNOS; increased HDL plasma levels; restored renal, hepatic and cardiac functions
Tanaka et al., 2020 [272]	Ten-week-old C57BL/6 mice. CSL-111 infusion 2 h post-sepsis. Three models (CLP; E. coli IAI76 intraperitoneal injection; P. aeruginosa pneumonia)	Improved survival rate in all the models. Decreased bacterial count at 24 h after CLP in both plasma and liver. Less lung inflammation in pneumonia model in treated mice. No significant differences in cytokine concentration in all the models.
Kwon et al., 2012 [273]	IP infusion of 4F peptide (10 mg/kg) 10 min after LPS challenge (10 mg/kg)	Increased lung S1P1 expression and phosphorylated Akt/Akt ratio in LPS-treated rats. Suppressed inhibitor κB-α degradation, NF-κB activation, E-selectin, and intercellular adhesion molecule-1 expression. Attenuated histologic damages in lung tissues. Improved survival rate.
Sharifov et al., 2013 [274]	Sprague-Dewey rats. IV infusion of L-4F 10 mg/kg 1 h post-LPS challenge (30 mg/kg)	L-4F significantly decreased mortality and reduces lung and liver injury, even when administered 1 hour post LPS
Mc Donald et al., 2003 [275]	Pretreatment with rHDL 5 minutes before LSP administration (6 mg/kg IV) in rats	Reduced histological tissue injury in the lung, liver, and intestine. Attenuated the expression of P-selectin and intercellular adhesion molecule-1 in the renal glomerulus. No effects in preventing the hypotension nor the rise in plasma levels of TNF-α (at 90 min)

## Data Availability

Not applicable.
